# Deletion of high-molecular-weight glutenin subunits in wheat significantly reduced dough strength and bread-baking quality

**DOI:** 10.1186/s12870-018-1530-z

**Published:** 2018-12-03

**Authors:** Yingjun Zhang, Mengyun Hu, Qian Liu, Lijing Sun, Xiyong Chen, Liangjie Lv, Yuping Liu, Xu Jia, Hui Li

**Affiliations:** 1 Institute of Cereal and Oil Crops, Hebei Academy of Agriculture and Forestry Sciences, 162 Hengshan Street, Shijiazhuang, 050035 China; 2 Institute of Genetics and Developmental Biology, Chinese Academy of Sciences, 1 Beichenxi Road, Beijing, 100101 China

**Keywords:** Common wheat, High-molecular-weight glutenin subunits, Post-transcriptional gene silencing, Glutenin macropolymer, Gliadin content, Dough quality

## Abstract

**Background:**

High-molecular-weight glutenin subunits (HMW-GS) play important roles in the elasticity of dough made from wheat. The HMW-GS null line is useful for studying the contribution of HMW-GS to the end-use quality of wheat.

**Methods:**

In a previous work, we cloned the *Glu-1E*^*b*^*x* gene from *Thinopyrum bessarabicum* and introduced it into the wheat cultivar, Bobwhite. In addition to lines expressing the *Glu-1E*^*b*^*x* gene, we also obtained a transgenic line (LH-11) with all the HMW-GS genes silenced. The HMW-GS deletion was stably inherited as a dominant and conformed to Mendel’s laws. Expression levels of HMW-GS were determined by RT-PCR and epigenetic changes in methylation patterns and small RNAs were analyzed. Glutenins and gliadins were separated and quantitated by reversed-phase ultra-performance liquid chromatography. Measurement of glutenin macropolymer, and analysis of agronomic traits and end-use quality were also performed.

**Results:**

DNA methylation and the presence of small double-stranded RNA may be the causes of post-transcriptional gene silencing in LH-11. The accumulation rate and final content of glutenin macropolymer (GMP) in LH-11 were significantly lower than in wild-type (WT) Bobwhite. The total protein content was not significantly affected as the total gliadin content increased in LH-11 compared to WT. Deletion of HMW-GS also changed the content of different gliadin fractions. The ratio of ω-gliadin increased, whereas α/β- and γ-gliadins declined in LH-11. The wet gluten content, sedimentation value, development time and stability time of LH-11 were remarkably lower than that of Bobwhite. Bread cannot be made using the flour of LH-11.

**Conclusions:**

Post-transcriptional gene silencing through epigenetic changes and RNA inhibition appear to be the causes for the gene expression deficiency in the transgenic line LH-11. The silencing of HMW-GW in LH-11 significantly reduced the dough properties, GMP content, wet gluten content, sedimentation value, development time and stability time of flour made from this wheat cultivar. The HMW-GS null line may provide a potential material for biscuit-making because of its low dough strength.

**Electronic supplementary material:**

The online version of this article (10.1186/s12870-018-1530-z) contains supplementary material, which is available to authorized users.

## Background

Wheat (*Triticum aestivum* L.) is a staple crop grown widely in the world as a source of flour for various kinds of foods due to the presence of gluten proteins in its seeds. Gluten is commonly classified into glutenins and gliadins. Gliadins are responsible for the extensibility and viscosity of dough [1]. Glutenins are of two major types: high-molecular-weight glutenin subunits (HMW-GS) and low-molecular-weight glutenin subunits (LMW-GS), both of which affect the strength and elasticity of wheat dough [2]. It was reported that HMW-GS constitute linear chains and protein networks, while LMW-GS exist as clusters and aggregates formed by branching from linear chains. Gliadins are equally spread throughout the dough, exhibiting ‘space-filling’ roles [3], whereas, the HMW-GS are the major factors affecting the end-use quality of wheat [1, 4].

The HMW-GS are encoded by *Glu-A1*, *Glu-B1,* and *Glu-D1* which are located at the *Glu-1* loci on the long arms of chromosomes 1A, 1B and 1D, respectively [5]. Each locus is comprised of two tightly linked genes encoding an x-type and a y-type subunit which have different electrophoretic mobilities [1]. In theory, there should be six HMW-GS (including 1Ax, 1Ay, 1Bx, 1By, 1Dx, and 1Dy) in hexaploid common wheat. Owing to the silencing of some HMW-GS genes, only three to five subunits are present in an individual common wheat variety [6]. For example, genes *1Bx*, *1Dx*, and *1Dy* are normally expressed, whereas *1Ay* is often not expressed in common wheat [7]. The HMW-GS have many repeat units such as nona- (GYYPTSL/PQQ), hexa- (PGQGQQ) and tri-peptides (GQQ) in the central repetitive domain. The central domain is flanked by two highly conserved non-repetitive N- and C-terminal domains that are rich in charged residues [8]. It is demonstrated that the central repetitive domain constitutes β-turns, while both the N- and C-terminal domains are rich in α-helices by molecular modeling and secondary structural analyses [9, 10]. Since the disulphide bonds between the cysteine residues affect the conformation and structure of the protein, the number and distribution of cysteines in each of the three domains of HMW-GS are particularly interesting. Most cysteines are in the terminal domains. Normally, there is only one conserved cysteine in the C-terminus, while there are three and five conserved cysteine residues in the N-terminal domain of the larger x-type subunits and the smaller y-type subunits, respectively [11]. These are the most crucial features of the glutenins associated with the physical properties of wheat dough [12].

The discovery of HMW-GS from wheat relative species not only enhances end-use quality but also broadens the genetic diversity. Many studies have focused on different landraces, wild species and wheat relatives [4, 13] because they provide abundant diversity of *Glu-1* loci in comparison with bread wheat. People have identified 22 alleles for *Glu-A1*, 52 for *Glu-B1* and 36 for *Glu-D1* based on the Grain Genes 2.0 database [7]. For example, the *Glu-R1* locus of rye [14], *Glu-E1* locus of *Elytrigia elongata* [15], *Glu-V1* locus of *Dasypyrum villosum* [16, 17], *Glu-U1* locus of *Aegilops umbellulata* [18] and *Glu-C1* locus of *Aegilops caudata* [19] have been presumed or confirmed to be the loci of interest encoding HMW-GS corresponding to wheat.

The combinations of HMW-GS subunits are thought to account for up to 70% of the good bread-making qualities of wheat [20, 21]. The *Glu-D1* locus has the largest effect on the rheological properties and dough quality of the wheat flour [22]. As an important breeding strategy, scientists try to aggregate superior HMW-GS together to improve wheat dough quality. The cultivars with a combination of *1Dx5* + *1Dy10* have suitable viscoelastic properties for good loaf volume [23, 24]. The 1Ax2^*^ at *Glu-A1* is related to greater dough strength and better bread-baking [25]. The *1Bx17* + *1By18*, *1Bx13* + *1By16* and *1Bx7* + *1By8* at *Glu-B1* show higher elastic moduli and viscosity coefficients which have positive effects on bread volume [26, 27]. However, their allelic variants such as *1Ax null, 1Bx6 + 1By8*, and *1Dx2 + 1Dy12* are associated with poor baking quality [28–31]. The effects of different subunits on dough quality may be due to their molecular weight and the number of cysteine residues. There are more cysteines in the y-type subunits than x-type, making y-type subunits more important for baking quality improvement because of their greater abilities to form inter- and intra-chain disulphide bonds [32]. An extra cysteine residue in the N-terminal domain of *1Dx5* enhances dough elasticity, whereas two less cysteines in *1Bx20* reduces wheat dough strength [4, 33, 34].

Each glutenin subunit accounts for about 2% of the total grain protein and the differences in gene expression could result in quantitative effects on total HMW-GS content, which in turn affects processing quality. For example, increasing the 1Dx5 or 1Dy10 subunits and the naturally duplicated *1Bx7* gene (*Bx7*^*OE*^) led to better dough strength [35, 36]. On the other hand, wheat lines with individual HMW-GS deficiencies at the *Glu-1* locus were characterized and used to determine the contributions of single HMW-GS on gluten micro structure, glutenin polymerization, dough mixing properties and bread-making quality [37–40]. However, the effect of silencing all the HMW-GS genes on wheat quality has not been studied. In previous work, we have obtained an HMW-GS null line, LH-11, which is of value for analyzing the contributions of HMW-GS to wheat flour processing quality. Therefore, in the current study we had the following objectives: (a) to find out the mechanism behind HMW-GS gene silencing in line LH-11 and (b) to evaluate the effects of deletion of HMW-GS on dough structure, gliadin fragments, agronomic traits and end-use quality of wheat.

## Results

### HMW-GS are silenced in transgenic line LH-11

**Fig. 1:**
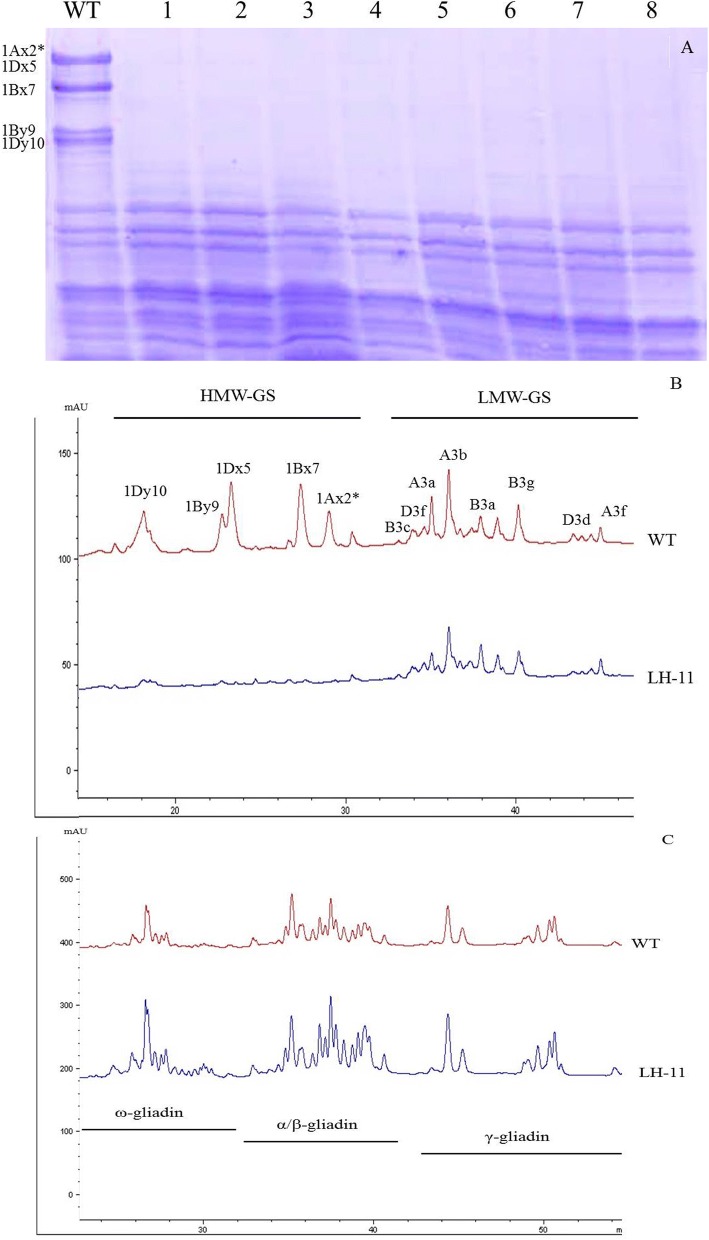
Comparison of glutenin subunits and gliadins in wild-type and transgenic line LH-11 detected by SDS-PAGE and RP-UPLC. **a** SDS-PAGE analysis of LH-11. WT, wild-type Bobwhite; 1–4, transgenic line LH-11 in T_1_ generation; 5–8, transgenic line LH-11 in T_2_ generation; (**b**) RP-UPLC analysis of glutenin subunits; (**c**) RP-UPLC analysis of gliadins

**Fig. 2:**
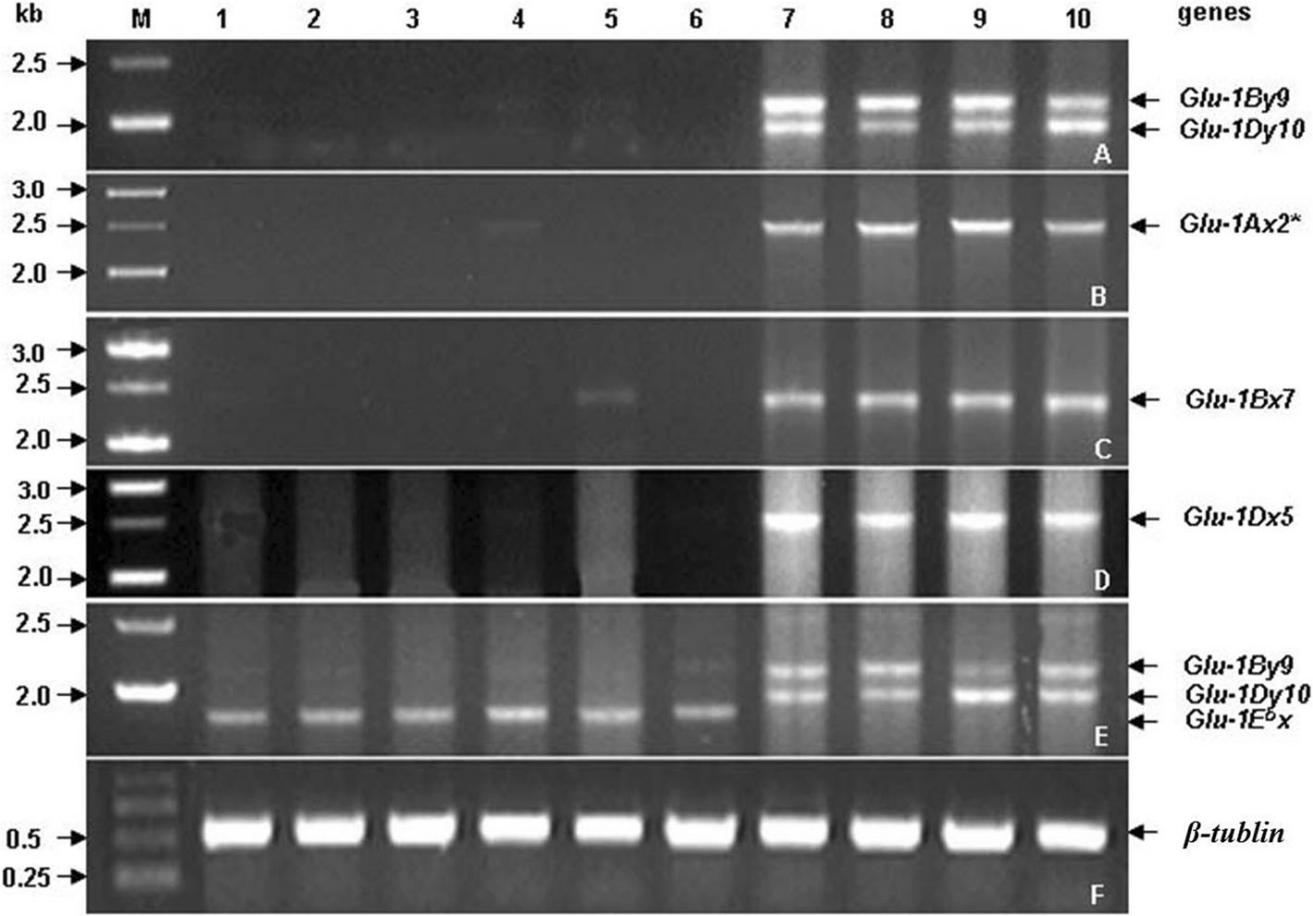
Expression analysis of HMW-GS genes (*Glu-1*) and *Glu-1E*^*b*^*x* gene using RT-PCR. Almost all the HMW-GS genes were silenced in transgenic line LH-11. **a** *Glu-1By9* and *Glu-1Dy10* genes. **b** Glu-1Ax2* gene. **c** *Glu-1Bx7* gene. **d** *Glu-1Dx5* gene. **e** *Glu-1By9*, *Glu-1By9,* and *Glu-1E*^*b*^*x* genes. **f** *β-tubulin*. M, marker; 1–6 cDNA from the seeds of transgenic line LH-11 at 6, 9, 12, 15, 18 and 21 days after flowering (DAF); 7–10 cDNA from the seeds of wild-type Bobwhite at 9, 12, 15 and 18 DAF. The numbers on the left side of the figure indicate the sizes (kb) of the PCR bands. The characters on the right side of the figure are the gene names

The spring wheat variety, Bobwhite, was transformed with the *Glu-1E*^*b*^*x* gene. We obtained ten positive transgenic lines expressing the *Glu-1E*^*b*^*x* gene and one transgenic line (LH-11) with all the HMW-GS silenced. None of the HMW-GS were detected in LH-11, including the 5 HMW-GS (1Ax2*, 1Bx7, 1By9, 1Dx5 and 1Dy10) of Bobwhite and the 1E^b^x of *Thinopyrum bessarabicum* as well, by sodium dodecyl sulphate polyacrylamide gel electrophoresis (SDS-PAGE) (Fig. 1a). RT-PCR was carried out to determine the expression level changes of HMW-GS genes (*Glu-1*) between wild-type Bobwhite and line LH-11. Total RNA was isolated from the seeds of LH-11 and wild-type Bobwhite at 6, 9, 12, 15, 18 and 21 days after flowering (DAF), reverse-transcribed to cDNA and amplified by PCR. The *β-tubulin* gene had the same PCR amplification level across all samples, indicating the cDNA of all the samples were at equal concentrations (Fig. 2). There were five HMW-GS, namely 1Ax2*, 1Bx7, 1By9, 1Dx5 and 1Dy10, in Bobwhite that were encoded by genes Glu-1Ax2*, *Glu-1Bx7*, *Glu-1By9*, *Glu-1Dx5*, and *Glu-1Dy10*, respectively. All five *Glu-1* genes were completely blocked in the seeds of LH-11 except weak signals of Glu-1Ax2* in seeds at 15 DAF and *Glu-1Bx7* in seeds at 18 DAF, whereas they were strongly expressed in seeds of wild-type Bobwhite. However, *Glu-1E*^*b*^*x* was expressed normally in LH-11. The expression of HMW-GS genes were obviously silenced or drastically inhibited by the *Glu-1E*^*b*^*x* gene in transgenic line LH-11.

### DNA methylation and small RNAs were involved in silencing of HMW-GS

**Fig. 3:**
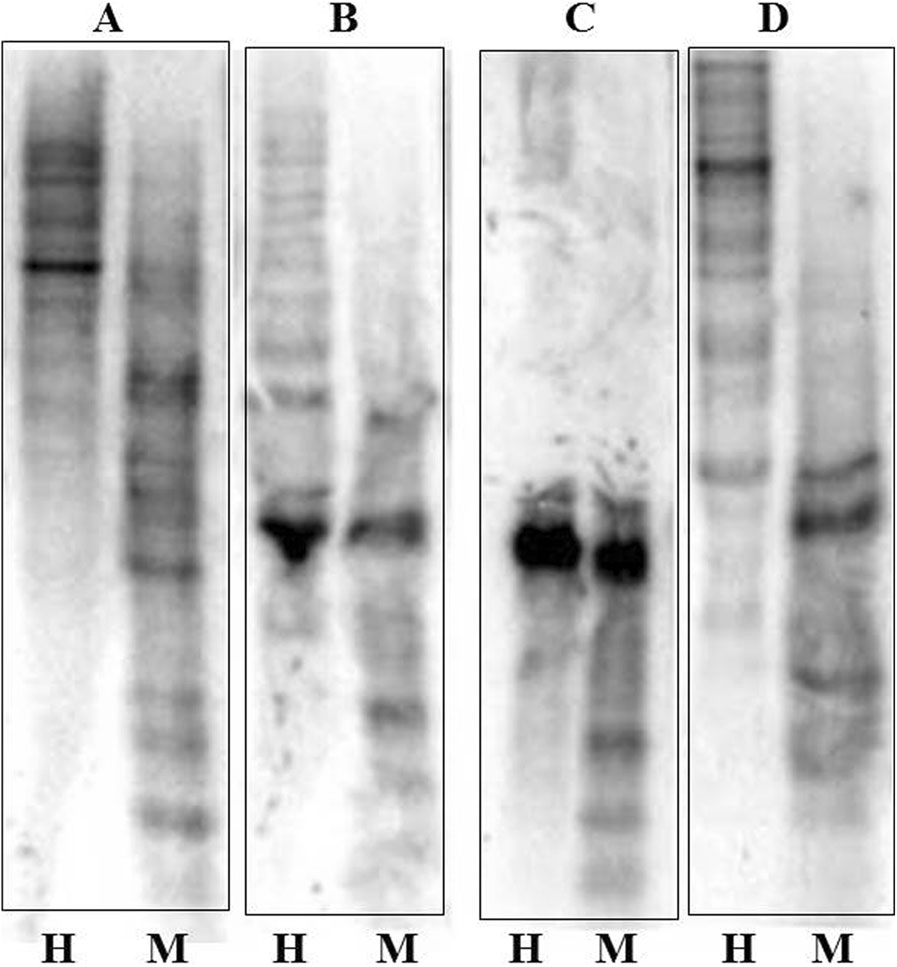
DNA methylation analysis of transgenic line LH-11. DNA methylations were detected in *Glu-1Bx7*, *Glu-1Dx5*, *Glu-1Dy10* and *Glu-1E*^*b*^*x* genes of LH-11. **a** *Glu-1Bx7* gene. H, *Hpa*II/*Hin*dIII digestion; M, *Msp*I/*Hin*dIII digestion. **b** *Glu-1Dx5* gene. H, *Hpa*II/*Hin*dIII digestion; M, *Msp*I/*Hin*dIII digestion. **c** *Glu-1Dy10* gene. H, *Hpa*II*/Nae*I digestion; M, *Msp*I/*Nae*I digestion. **d** *Glu-1E*^*b*^*x* gene. H, *Hpa*II/*Eco*RI digestion; M, *Msp*I/*Eco*RI digestion

**Fig. 4:**
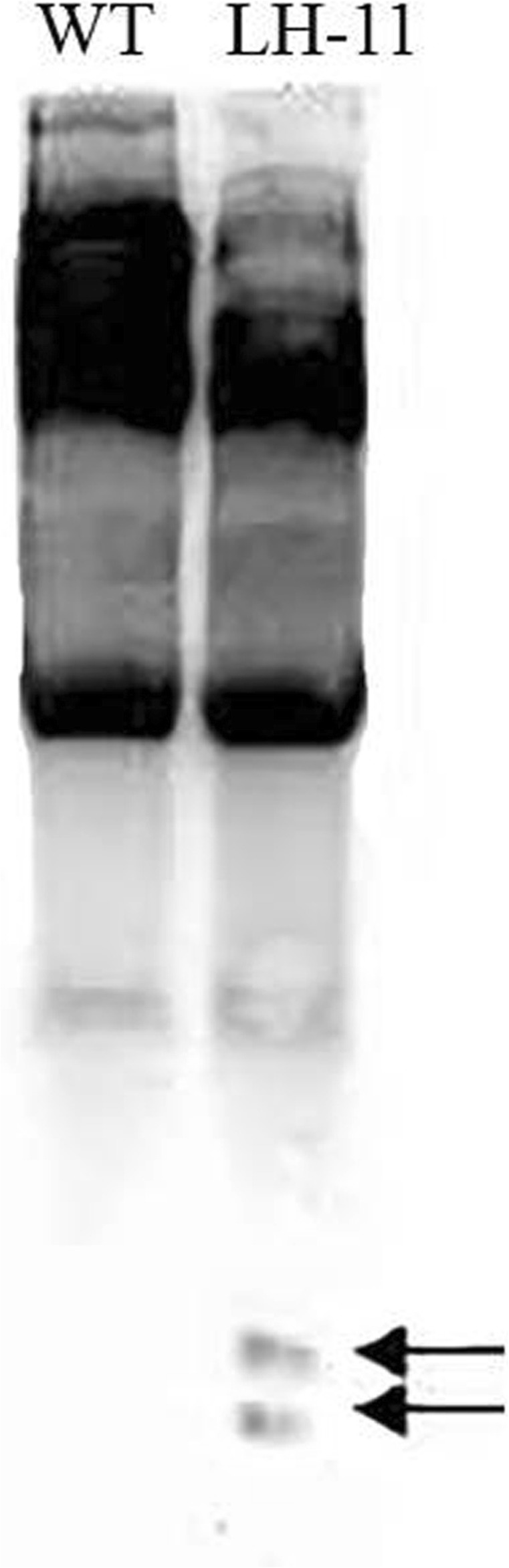
Small RNA analysis of LH-11. Two hybridization signals of small RNA (about 20–25 nt in size) were detected in LH-11, whereas no signal in wild-type Bobwhite. Arrows point to the fragments of small RNA

In order to find out what caused silencing of HMW-GS in LH-11 seeds, we performed analyses for DNA methylation and small RNAs. DNA methylations were detected in the *Glu-1Bx7*, *Glu-1Dx5*, *Glu-1Dy10* and *Glu-1E*^*b*^*x* genes of LH-11. The four genes showed different banding patterns when digested with *Hpa*II or *Msp*I (Fig. 3). DNA was cut more thoroughly with *Msp*I and smaller fragments were achieved with *Msp*I than with *Hpa*II, demonstrating that all four genes had significant DNA methylations. We then selected the *Glu-1Dy10* gene, which had the lowest RNA expression level as an example to carry out small RNA analysis. Two hybridization signals of small RNAs were detected in LH-11, whereas no signals were detected in Bobwhite (Fig. 4). The lengths of the two small RNAs were 20–25 nt; so, it seemed that the silencing of HMW-GS in transgenic line LH-11 was caused by both DNA methylation and small RNAs.

### Deletion of HMW-GS in LH-11 is inherited like a dominant trait

To study the inheritance of line LH-11, we crossed LH-11 with five Chinese cultivars (Jinfeng5365, Gao8901, Luozhen1, Gao9411, and Kenong122), respectively. SDS-PAGE was performed to analyze the HMW-GS in F_1_ generation progenies. No HMW-GS were detected in any of the F_1_ generation progenies. The F_1_ was self-crossed to give rise to an F_2_ generation. Of these offspring, about 3/4 had no HMW-GS while 1/4 had HMW-GS; the ratio of segregation was 3:1 (Additional file 1: Table S1). The results showed that HMW-GS gene silencing was dominantly controlled and stably inherited in progenies according to a Mendelian pattern.

### Silencing of HMW-GS directly affected the accumulation of glutenin macropolymer (GMP) in LH-11 during seed development

**Fig. 5:**
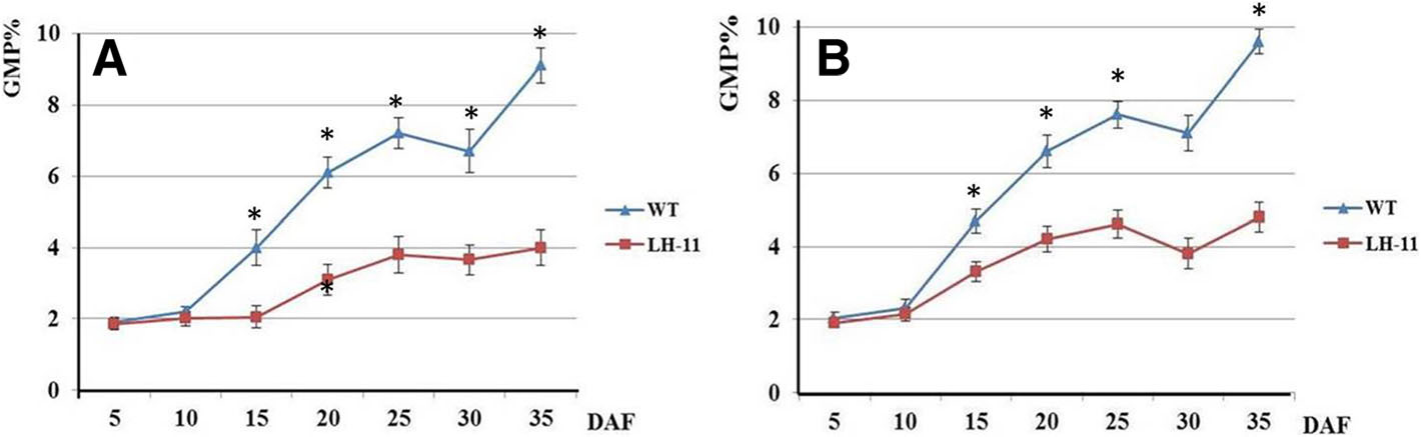
Accumulations of GMP during seed development in wild-type Bobwhite and transgenic line LH-11. **a** year 2008; **b** year 2009. WT, wild-type Bobwhite. Statistical significance was determined by a Student’s *t*-test at *P* < 0.01

Seeds at different development stages of 5, 10, 15, 20, 25, 30 and 35 DAF were taken to carry out GMP analysis. The accumulation of GMP showed a regular increase during seed development (Fig. 5). After slow growth in the early stage of seed development (from 5 to 10 DAF), GMP content increased rapidly from 10 to 25 DAF, slightly decreased from 25 to 30 DAF, and reached its highest value at the mature stage. The GMP content of Bobwhite was similar to that of LH-11 during the early development period (from 5 to 10 DAF), whereas at the two rapid accumulation stages (10–25 DAF and 30–35 DAF), the accumulation rate of GMP in LH-11 was significantly lower than that in Bobwhite. Furthermore, the final content of GMP in LH-11 was much lower than that in Bobwhite-about half.

### Total gliadin content and proportion of ω-gliadin were increased in the LH-11 line

There was no difference in total protein content between LH-11 and Bobwhite (Table 1). Reversed-phase ultra-performance liquid chromatography (RP-UPLC) was employed to determine the effects of the absence of HMW-GS on LMW-GS and gliadin content. All the HMW-GS were thoroughly silenced (Fig. 1b). The peak area of LMW-GS in LH-11 (2714.4 ± 46.2) was decreased compared to Bobwhite (3127.6 ± 51.3), whereas total gliadin content significantly increased in LH-11 (Fig. 1c). Deletion of HMW-GS also caused changes in the percentage content of different gliadins fragments. The ratio of ω-gliadin increased from 20.5 to 25.8%, however, α/β-gliadin and γ-gliadin declined from 54.3 to 50.7% and 25.2 to 23.5%, respectively (Table 2).

**Table 1:** Rheological analysis of dough from transgenic line LH-11

Material	TP%	WG%	SV (ml)	WA%	DT (min)	ST (min)	BV (ml)	BS
Bobwhite	15.4 ± 0.2	31.0 ± 1.6*	28.8 ± 0.8*	61.8 ± 1.7*	6.0 ± 0.3*	7.0 ± 0.3*	770.0 ± 23.2*	74.0 ± 2.2*
LH-11	15.0 ± 0.3	3.4 ± 0.2	6.8 ± 0.2	55.0 ± 1.4	0.4 ± 0.1	0.6 ± 0.1		

* Statistical significance was determined by a Student’s *t*-test at *P* < 0.01

*TP* total protein content, *WG* wet gluten content, *SV* sedimentation value, *WA* water absorption, *DT* development time, *ST* stability time, *BV* bread volume, *BS* bread score

**Table 2:** Relative content of glutenins and gliadins by RP-UPLC

	HMW-GS%	LMW-GS%	Glutenins^a^	ω-gliadin%	α/β-gliadin%	γ-gliadin%	Gliadins^a^
Bobwhite	48.8 ± 1.2**	51.2 ± 0.9**	6112.3 ± 128.3**	20.5 ± 0.7*	54.3 ± 1.3*	25.2 ± 0.5*	17,775.0 ± 689.3**
LH-11	0	100	2714.6 ± 46.2	25.8 ± 1.1	50.7 ± 1.4	23.5 ± 0.6	30,017.1 ± 942.2

^a^ The peak area (1000 uV/S) of total glutenins and gliadins*Statistical significance was determined by a Student’s *t*-test at *P* < 0.05** Statistical significance was determined by a Student’s *t*-test at *P* < 0.01

### Plant height and seed number increased in LH-11

To investigate the effects of deletion of HMW-GS on agronomic traits of LH-11, we measured the plant height, panicle number, and seed number among other factors (Table 3). The plant height, spike length, seeds per panicle and seeds per plant in LH-11 were significantly greater than in wild-type Bobwhite. The height of LH-11 increased by 7% and the seed numbers per plant increased drastically from 160.5 in Bobwhite to 198.8 in LH-11 (23.9% increase). The panicle and tiller numbers per plant increased slightly, whereas the floret numbers decreased slightly in LH-11, but the differences did not reach a significant level (*P* < 0.05).

**Table 3:** Agronomic traits of LH-11 and Bobwhite

Material	Plant height	Panicles per plant	Tiller number	Spike length	Floret number	Seeds per panicle	Seeds per plant
LH-11	74.79 ± 1.5*	3.3 ± 0.4	5.1 ± 0.2	10.5 ± 0.4*	81.9 ± 7.5	59.9 ± 4.1*	198.8 ± 4.9*
Bobwhite	69.4 ± 1.2	3.0 ± 0.3	4.9 ± 0.8	9.9 ± 0.1	83.6 ± 2.2	53.6 ± 1.4	160.5 ± 7.7

* Statistical significance was determined by a Student’s *t*-test at *P* < 0.05

### Bread-baking quality of LH-11 was significantly reduced

The differences in rheological and farinograph properties of dough from LH-11 compared to Bobwhite were measured. Wet gluten content, sedimentation value, water absorption, development time and stability time in LH-11 decreased significantly (*P* < 0.01) compared to Bobwhite. The wet gluten content in LH-11 was reduced so much (from 31.0 to 3.4%) that the development time and stability time were very short: the development time decreased from 6.0 to 0.4 min, and the stability time from 7.0 to 0.6 min (Table 1). Bread cannot be made successfully from the flour of LH-11.

## Discussion

It has been accepted that the variation in HMW-GS composition strongly affects wheat processing quality. Lines exhibiting no expression of HMW-GS such as LH-11 described here can provide wheat breeders with new materials to study end-use functionality. In this study, a transgenic line LH-11 with all the HMW-GS silenced was obtained in addition to ten positive transgenic lines expressing the *Glu-1E*^*b*^*x* gene. In LH-11, none of the HMW-GS including the five endogenous HMW-GS of the donor plant, Bobwhite, and the 1E^b^x of *Th. Bessarabicum* were detectable by SDS-PAGE (Fig. 1a). Our results showed that LH-11 was a stable line and the trait of deletion of HMW-GS was inherited by the progenies. LH-11 was crossed with five Chinese wheat cultivars. All the F_1_ seeds and ¾ of the F_2_ seeds had deletions of HMW-GS (Additional file 1: Table S1), showing that it followed Mendel’s dominant gene inheritance law.

In transgenic line LH-11, *Glu-1E*^*b*^*x* was transcribed successfully into RNA, but it was not translated into protein. All of the five homologous endogenous *Glu-1* genes (Glu-1Ax2*, *Glu-1Bx7*, *Glu-1By9*, *Glu-1Dx5* and *Glu-1Dy10*) in Bobwhite were degraded at the RNA level (Fig. 2), which meant that post-transcriptional gene silencing (PTGS) was triggered in LH-11. PTGS is thought to be a universal gene regulation system in biological processes including defense against viruses and regulation of gene expression [41]. PTGS mostly occurs when the exogenous gene is homologous to the endogenous gene [42]. This phenomenon was first discovered in 1990 and is also called ‘co-suppression’ because the expression of both the introduced and the homologous endogenous genes were suppressed together [43, 44]. Because of co-suppression, silencing of endogenous HMW-GS after transformation has been commonly detected in wheat lines that contain HMW-GS transgenes designed for over-expression [45–47]. We postulated that PTGS was occurring in LH-11 either because of DNA methylation or the presence of small, double-stranded (ds) RNAs.

There are two main mechanisms for how DNA methylation inhibits gene expression. First, modification of cytosine bases can directly prevent transcription factors from binding to DNA recognition sequences [48, 49]. Second, DNA methylation results in chromatin modification and remodeling through the action of methyl-cytosine binding proteins (MBPs) and histone deacetylases [50, 51]. Here we showed that there were different degrees of DNA methylation in four genes *Glu-1Bx7*, *Glu-1Dx5*, *Glu-1Dy10* and *Glu-1E*^*b*^*x*, indicating that DNA methylation may cause gene silencing in LH-11 (Fig. 3). Double-stranded RNA is another trigger of PTGS. Plants can recognize dsRNAs from transgenes or viruses and cut them into short RNAs (21–26 nt) such as small interfering RNAs (siRNAs) and microRNAs (miRNAs) with an enzyme called Dicer [52–55]. The miRNAs and siRNAs are incorporated into the RNA-induced silencing complex (RISC) resulting in transcript cleavage [56, 57]. Researchers have detected significant accumulations of siRNAs in various PTGS systems in plants [58], so endogenous small RNAs may also play key roles in regulating gene expression and causing PTGS [59]. We isolated total RNA from T_4_ generation seeds of LH-11 and separated the small RNAs. Northern blots using *Glu-1Dy10* RNA as probe gave two hybridization signals of small RNAs in LH-11, whereas no signal was detected in wild-type Bobwhite (Fig. 4). Thus, small RNAs may be another way that HMW-GS are silenced in LH-11.

Although they represent only 10% of wheat storage proteins, HMW-GS have been recognized as crucial factors in determining the viscoelastic properties of wheat dough [60]. The physical properties of dough stem from interactions between HMW-GS and other grain storage proteins via both inter- and intra-chain disulphide bonds forming glutenin macropolymers (GMP) which contribute to the elasticity and strength of dough [4]. It has been reported that the x- and y-type HMW-GS are linked via head-to-tail disulphide bonds to form a backbone of the polymer. The LMW-GS constitute branch points of the y-type subunits at four positions [61]. Because the cysteine residues of HMW-GS affect polymeric behavior [9, 62], the composition and quantity of HMW-GS significantly affect the particle size and amount of GMP in flour [63]. Loss of HMW-GS from the polymer is always consistent with the time of dough breakdown. [1]. In this study, we analyzed the dynamic change of GMP at different seed development stages of the wild-type Bobwhite and transgenic line LH-11. The accumulation rate of GMP in LH-11 was significantly lower than that in Bobwhite at 10–25 and 30–35 DAF, which resulted in the final content of GMP in LH-11 being only half of that in Bobwhite (Fig. 5). Because HMW-GS is necessary for the formation of the dough protein network, the absence of HMW-GS resulted in the formation of ‘sheets’ in dough rather than a three-dimensional structure [64]. The decrease in GMP content may be one of the reasons for the decline in wheat flour quality of LH-11.

In addition to the HMW-GS, gliadins also play important roles in determining end-use wheat quality. Gliadins account for about 50% of seed storage proteins and generally contribute to the extensibility and viscosity of wheat dough [65]. The gliadins are divided into three types: α/β-, γ- and ω-gliadins [66]. Unlike glutenins which form polymers by both inter- and intra-chain disulphide bonds, gliadins are monomeric proteins that contain only intra-chain bonds (Shewry and Halford, 2003). Differences in the disulphide bonding properties of glutenins and gliadins affect how they establish the GMP and gluten structures. Our results showed that silencing of HMW-GS increased the total gliadin content in LH-11 (Table 2). It is generally agreed that total gliadin content has a significant negative correlation with dough properties such as development time and stability time [67]. In the present study, we checked gliadin levels in LH-11 by RP-UPLC and found that increase in total gliadin content may be another reason of bread-baking quality breakdown besides the absence of HMW-GS in LH-11. Deletion of HMW-GS also caused changes in the percentage content of different gliadins fractions. The ratio of ω-gliadin increased, whereas α/β- and γ-gliadins declined in LH-11 (Table 2). Different types of gliadins have different effects on wheat quality depending on their properties. The ω-gliadins lack cysteine and cannot form disulphide bonds. The α/β-gliadins contain six cysteine residues and γ-gliadins contain eight cysteine residues [66]. Furthermore, ω-gliadins have a β-turn structure, while α/β- and γ-gliadins have a high proportion of α-helical and β-sheet structures [9]. The ω-gliadins are sulphur-poor, while, α/β- and γ-gliadins are sulphur-rich protein. Some studies indicated that α/β-gliadins and γ-gliadins were positively associated with loaf volume and development time, respectively [67, 68]. The increase in the proportion of ω-gliadins and decrease in both α/β- and γ-gliadins in LH-11 also reduced dough quality. The total protein content was not significantly affected in LH-11 comparing to Bobwhite (Table 1). The reduction of glutenins was compensated for by increasing gliadin content in the grain, suggesting that wheat has a good system for balancing gluten proteins [69]. The wet gluten content and sedimentation value in LH-11 were much lower than that of the wild-type (Table 1). Development time and stability time are closely linked to dough strength. Results reported in the present study showed that the average development time and stability time in LH-11 were remarkably lower than in Bobwhite (Table 1). The flour of LH-11 is unsuitable for bread-making, but has great potential for making biscuits because of its low dough strength.

## Conclusions

In the transgenic wheat line LH-11, all the HMW-GS were silenced and this genetic modification was stably passed on to progenies by crossing LH-11 with other wheat cultivars. We found DNA methylations and small RNA signals in HMW-GS genes of LH-11, indicating that DNA methylation and double-stranded RNA may be the reasons for post-transcriptional gene silencing in LH-11. The silencing of HMW-GS in LH-11 significantly altered its dough properties. The accumulation rate of GMP at the rapid accumulation stages (10–25 DAF and 30–35 DAF) and final content of GMP in LH-11 were much lower than in wild-type Bobwhite. The content of LMW-GS decreased whereas total gliadin content significantly increased in LH-11 compared to the wild-type. Deletion of HMW-GS also caused changes in the percentage content of different gliadins fragments. The ratio of ω-gliadin increased from 20.5 to 25.8%, however, α/β-gliadin and γ-gliadin declined from 54.3 to 50.7% and 25.2 to 23.5%, respectively. The wet gluten content and sedimentation value of LH-11 were remarkably lower than that of Bobwhite. The development time decreased from 6.0 to 0.4 min and the stability time from 7.0 to 0.6 min. Therefore, flour from LH-11wheat has good potential for biscuit-making because of its low dough strength.

## Methods

### Plant materials

In a previous study, we cloned the *Glu-1E*^*b*^*x* gene (GenBank accession AY525782) encoding HMW-GS of *Th. Bessarabicum* and introduced it into the common wheat cultivar, Bobwhite, using a biolistic transformation method. Besides ten transgenic events characterized by expression of the *Glu-1E*^*b*^*x* gene, we also, fortunately obtained a transgenic line, LH-11, with all HMW-GS silenced. LH-11 is in the T_6_ generation now and the trait of deletion of all HMW-GS is still stably inherited. To study the genetic inheritance of LH-11, we crossed it with five Chinese wheat cultivars (Jinfeng5365, Gao8901, Luozhen1, Gao9411, and Kenong122), respectively. The F_1_ was self-crossed to give rise to F_2_ generation. The field trials in the present study were carried out in randomized complete blocks with three replicates at Shijiazhuang, Hebei province, China.

### Analysis of expression levels of HMW-GS genes in LH-11

Total RNA was isolated (three biological replicates) from the seeds of positive transgenic lines and wild-type Bobwhite at 6, 9, 12, 15, 18 and 21 days after flowering (DAF) using the Trizol method (www.tiangen.com). All samples were DNase-treated before reverse transcription. The first-strand cDNA was synthesized by MMLV reverse transcriptase (http://www.promega.com.cn) using oligo(dT) as a primer. Reverse transcriptional products were adjusted to an equal concentration according to the PCR signal generated from the internal standard house-keeping gene, *β-tubulin*, and used as templates for RT-PCR. The primers used in RT-PCR are listed in Table 4. RT-PCR was performed in total volumes of 20 μl, including 2 μl of 10× LaTaq buffer, 0.5 μl of dNTP (2.5 mM of each dNTP), 1 μl of each primer (5 μM), 1 U of La DNA polymerase and 80 ng of template cDNA. PCR conditions were: initial denaturation at 94 °C for 3 min, followed by 40 cycles at 94 °C for 30 s, 58 °C for 30 s and 72 °C for 3 min, and a final extension for 5 min at 72 °C. RT-PCR products were separated in 1% agarose gels, and the bands were visualized with ethidium bromide.

**Table 4:** Primer sets used in this study

Primer set	Sequence 5′-3’	Amplified target
Ax	F: AGATGACTAAGCGGTTGGTTC	The genes of x-type HMW-GS on *Glu-A1* locus
	R: CTGGCTGGCCAACAATGCGT	
Bx	F: ATGGCTAAGCGCCTGGTCCT	The genes of x-type HMW-GS on *Glu-B1* locus
	R: TGCCTGGTCGACAATGCGTGC	
Dx	F: ATGGCTAAGCGGTTAGTCCT	The genes of x-type HMW-GS on *Glu-D1* locus
	R: CTGGCTGGCCGACAATGCGT	
Y-type	F: ATGGCTAAGCGGTTGGTCCT	The genes of y-type HMW-GS
	R: GGCTAGCCGACAATGCGTCG	
Tublin	F: GGCTAGCCGACAATGCGTCG	*β-tubulin* gene of wheat
	R: GGCTAGCCGACAATGCGTCG	

### DNA methylation analysis

DNA methylation analyses in this study relied on digestion with methylation-sensitive restriction enzymes followed by gel electrophoresis and hybridization on southern blots. Restriction enzymes *Msp*I and *Hpa*II have the same recognition site CCGG. *Hap*II is a methylation-sensitive restriction enzyme which is inhibited by 5^me^C in the sequence context CpG, whereas its isoschizomer *Msp*I is not inhibited by CpG methylation. The patterns of cutting by these two enzymes can provide a read-out of DNA methylation. In T_4_ generation, we chose four genes *Glu-1Bx7*, *Glu-1Dx5*, *Glu-1Dy10* and *Glu-1E*^*b*^*x* for DNA methylation examination. By analyzing gene sequences of these four genes, we selected different restriction enzymes to do double digests of different genes; *Hin*dIII + *Hpa*II/*Msp*I were employed to digest *Glu-1Bx7* and *Glu-1Dx5*, *Nae*I + *Hpa*II/*Msp*I were used to digest *Glu-1Dy10* and *EcoR*I + *Hpa*II/*Msp*I were used to cut *Glu-1E*^*b*^*x*, respectively. Genomic DNA (200–500 ng) was cleaved with corresponding restriction enzymes such as *Hin*dIII + *Hpa*II or *Hin*dIII + *Msp*I in two separate reactions. Then, the digestion products were separated by electrophoresis on 0.8% agarose gel and hybridized using [α-^32^P] dCTP-labelled gene fragment as probes (Additional file 2: Table S2).

### Small RNA detection

Small RNA extraction was performed using the method reported by Peng et al. [70] with minor modifications. Total RNA was isolated from immature T_4_ generation seeds of line LH-11 using TRNzol reagent (http://www.tiangen.com/en/). Samples frozen in liquid nitrogen were ground to a fine powder with a mortar and pestle. About 100 mg of powder was transferred into a 2 ml centrifuge tube containing 1 ml of TRNzol. After being thoroughly mixed by vortexing, the mixture was kept at room temperature for 10 min. Then, 0.2 ml of chloroform was added, the tubes were vortexed vigorously and the mixture was centrifuged at 12,000 rpm for 10 min at 4 °C. The upper aqueous phase was transferred to a new centrifuge tube and an equal volume of precipitation buffer (20% *w*/*v* PEG 8000, 1 M NaCl) was added. The tubes were incubated at 65 °C for 15 min, kept at room temperature for 10 min, and chilled on ice immediately for 40 min to precipitate the high molecular weight RNAs. Following centrifugation at 12,000 rpm for 10 min at 4 °C, the supernatant was collected as the fraction enriched in small RNAs. Small RNAs were precipitated with 1/10 volume of 3 M sodium acetate (pH 5.2) and 2.5 volume of precooled absolute ethanol at -20 °C overnight. The pellet was collected by centrifugation at 12,000 rpm for 20 min and rinsed twice with 80% ethanol. Small RNA detection was performed on gene *Glu-1Dy10* which was inhibited more thoroughly. Northern blot analysis was carried out according to a standard protocol using [α-32P]dCTP-labelled *Glu-1Dy10* RNA as a probe.

### Reversed-phase ultraperformance liquid chromatography (RP-UPLC) analysis

HMW-GS, LMW-GS, and gliadins were extracted from LH-11 (T_5_ generation) and wild-type Bobwhite with three biological replicates using published methods [71–73]. The quantitative analyses of glutenins and gliadins were made on an Acquity UPLC (Waters Corp.) with a Waters 300SB C18 column (50 × 2.1 mm i.d., 1.7 μm). The separation of glutenins was based on the program reported by Yu et al. [71]. The four eluants were: A, ultrapure water containing 0.06% (*v*/v) trifluoroacetic acid (TFA); B, acetonitrile (ACN) containing 0.06% TFA; C, ultrapure water; and D, methanol. The column was first balanced by increasing the concentration of B from 21 to 47% in 15 min. The ratios of A to B for weak washing and strong washing needles were 79:21 and 53:47%, respectively. The sample was washed with A from 95 to 5% and B from 5 to 95% in 5 min. Final washing was done with solution C from 90 to 10% and D from 10 to 90% three times within 30 min. The separation conditions of gliadins were taken from the method reported by Han et al. [74]. Two elution buffers were used: solution A was 0.06% TFA in ultrapure water and solution B was 0.06% TFA in ACN. The gradient program was set as solution B from 21 to 46%. The differentiations of glutenin and gliadin fractions were based on their elution characteristics. The relative content of each fragment was calculated according to its peak area.

### Glutenin macropolymer, agronomic traits, and end-use quality analysis

Transgenic line LH-11 and wild-type Bobwhite were planted and grown in a completely randomized block design at Shijiazhuang, Hebei province. In the years 2007 through 2008 (T_3_ generation) and 2008 through 2009 (T_4_ generation), seeds at different development stages (5, 10, 15, 20, 25, 30 and 35 days after flowering (DAF)) were taken to carry out glutenin macropolymer (GMP) analysis according to the method described by Don et al. [75]. Observations on growth and yield-contributing traits (T_3_ generation) such as plant height, number of spikes, number of seeds per plant, etc., were recorded for ten individuals. Dough rheological and farinograph properties of T_5_ generation seeds were used to evaluate the end-use quality. Data were statistically analyzed to find differences between transgenic and wild-type plants using Student’s *t*-test. All the tests were performed on three replicates.

## Additional files

Additional file 1: **Table S1.** Segregation of HMW-GS deletion trait in F_1_ and F_2_ generation offsprings of LH-11. (DOCX 16 kb)

Additional file 2: **Table S2.** The probe regions for different genes used for DNA methylation analysis. (DOCX 14 kb)

## Abbreviations

DAF: Days after flowering
GMP: Glutenin macropolymer
HMW-GS: High-molecular-weight glutenin subunits
LMW-GS: Low-molecular-weight glutenin subunits
PTGS: Post-transcriptional gene silencing
RP-UPLC: Reversed-phase ultra-performance liquid chromatography
RT-PCR: Reverse transcription-polymerase chain reaction
SDS-PAGE: Sodium dodecyl sulphate polyacrylamide gel electrophoresis
WT: Wild-type
