# Supplementary material for: Deletion of high-molecular-weight glutenin subunits in wheat significantly reduced dough strength and bread-baking quality
Source: BMC Plant Biol. 2018 Dec 3;18:319. doi: 10.1186/s12870-018-1530-z (PMC6276161; doi:10.1186/s12870-018-1530-z)
Supplement: Supplementary file 1 — Table S1. Segregation of HMW-GS deletion trait in F1 and F2 generation offsprings of LH-11. (DOCX 16 kb) [file 12870_2018_1530_MOESM1_ESM.docx]

**Table S1** Segregation of HMW-GS deletion trait in F_1_ and F_2_ generation offsprings of LH-11

| Cross | F_1_ generation | | | F_2_ generation | | | |  | |  |
| --- | --- | --- | --- | --- | --- | --- | --- | --- | --- | --- |
|  | **HMW-GS Deletion** | **HMW-GS Expression** | **Ratio** | **HMW-GS Deletion** | **HMW-GS Expression** | | **Ratio** | |  | |
| Jinfeng5356 × LH-11 | 22 | 0 | 22:0 | 46 | 15 | ~3:1 | |  | |  |
| Gao8901 × LH-11 | 24 | 0 | 24:0 | 24 | 8 | 3:1 | |  | |  |
| Luozhen1 × LH-11 | 35 | 0 | 35:0 | 24 | 8 | 3:1 | |  | |  |
| Gao9411 × LH-11 | 16 | 0 | 16:0 | 18 | 6 | 3:1 | |  | |  |
| Kenong122 × LH-11 | 31 | 0 | 31:0 | 24 | 8 | 3:1 | |  | |  |
