# Supplementary material for: Deletion of high-molecular-weight glutenin subunits in wheat significantly reduced dough strength and bread-baking quality
Source: BMC Plant Biol. 2018 Dec 3;18:319. doi: 10.1186/s12870-018-1530-z (PMC6276161; doi:10.1186/s12870-018-1530-z)
Supplement: Supplementary file 2 — Table S2. The probe regions for different genes used for DNA methylation analysis. (DOCX 14 kb) [file 12870_2018_1530_MOESM2_ESM.docx]

**Table S2** The probe regions for different genes for DNA methylation analysis

| Genes | Target region | Probe region |
| --- | --- | --- |
| *Glu-1Bx7* | coding sequence | 359-2494 |
| *Glu-1Dx5* | coding sequence | 387-2644 |
| *Glu-1Dy10* | promoter | (-2071)-128 |
| *Glu-1E^b^x* | coding sequence | 1-1818 |
